# Efficacy of Antimicrobial Dry Fog in Improving the Environmental Microbial Burden in an Inpatient Ward

**DOI:** 10.3390/antibiotics13121187

**Published:** 2024-12-06

**Authors:** Yashar Jalali, Andrea Kološová, Karol Džupa, Pavol Pavlovič, Monika Jalali, Peter Rácek, Nikola Zicháčková, Ján Kyselovič, Adriana Vasiková, Klaudia Glodová, Juraj Payer

**Affiliations:** 1 Faculty of Medicine, Comenius University in Bratislava, 5th Department of Internal Medicine, University Hospital Bratislava, Ružinov, Špitálska 24, 813 72 Bratislava, Slovakia, and Ružinovská 4810/6, 821 01 Bratislava, Slovakia; adamcova.monika@gmail.com (M.J.); jan.kyselovic@fmed.uniba.sk (J.K.); adrianavasikova@gmail.com (A.V.); glodova@ru.unb.sk (K.G.); juraj.payer@fmed.uniba.sk (J.P.); 2 Department of Hospital Hygiene and Epidemiology, University Hospital Bratislava, Ružinov, Ružinovská 4810/6, 821 01 Bratislava, Slovakia; kolosova@ru.unb.sk (A.K.); peter.racek@ru.unb.sk (P.R.);; 3 Detectair Technologies s.r.o., Trojičné námestie 7, 821 06 Bratislava, Slovakia; dzupa@detectair.sk (K.D.); pavlovic@detectair.sk (P.P.)

**Keywords:** dry fog, hospital infection control, hospital environmental bacterial burden, health-associated infections, multi-drug-resistant bacteria, airborne infections

## Abstract

**Background/Objectives:** In healthcare environments with high microbial loads, effective infection control measures are critical for reducing airborne and surface contamination. One of the novel modalities in the achievement of these goals is the use of antimicrobial mists, such as droplets, in the form of dry fog. Although the usage of dry fog in the disinfection of contained healthcare microenvironments is well known, the effect of such a system in terms of a meaningful reduction in the microbial burden in an open inpatient ward is unclear. Our objective was to assess the impact of scheduled dry fogging on microbial reduction in such settings. **Methods:** We collected air and surface samples from rooms receiving daily, biweekly, or no fogging (controls) over six months, establishing the baseline contamination and evaluating the reduction trends in treated rooms. The “reduction effect” was measured by tracking microbial isolation trends before and after treatment, while the “degree of reduction” assessed differences across rooms with varied disinfection schedules. **Results:** The results indicate that scheduled dry fogging significantly reduced microbial loads in treated rooms, especially with daily disinfection (SE = 64.484, *p* = 0.002). The airborne contamination in treated rooms showed a strong downward trend over time (SE = 19.192, *p* < 0.001). Surface contamination remained challenging due to frequent recontamination; however, treated rooms exhibited a consistent reduction in microbial presence (SE = 2.002, *p* = 0.010), confirming dry fogging’s role as a valuable adjunct to routine cleaning. **Conclusions:** In conclusion, this study highlights that dry fogging effectively reduces microbial loads in open, high-traffic healthcare environments, supporting its use as part of a multimodal infection control strategy.

## 1. Introduction

Nosocomial infections, or healthcare-associated infections (HAIs), pose a significant challenge to healthcare systems worldwide [1]. These infections can lead to prolonged hospital stays, increased morbidity and mortality, and substantial financial burdens [2,3]. The transmission of pathogens within healthcare environments is facilitated by various factors, including contaminated surfaces, airborne particles, and healthcare workers [4,5,6]. The emergence of multi-drug-resistant (MDR) bacteria has further exacerbated the problem, limiting the effectiveness of traditional antibiotic treatments [6,7,8].

The global spread of MDR bacteria has created an urgent need for innovative disinfection strategies [7,9]. The limited effectiveness of antibiotics against these pathogens often leads to severe, life-threatening infections that are difficult to treat [1,7,10]. The overuse and misuse of antibiotics have contributed to the development of MDR bacteria, making it imperative to explore alternative approaches to infection control [7,11,12].

One promising strategy for addressing the challenges posed by HAIs and MDR bacteria is to decrease or to eliminate the exposure of patients to these microorganisms by improving environmental disinfection [12]. One such strategy is the use of dry fog technology for indoor space and air disinfection [13]. Dry fog systems generate a fine mist of disinfectant that can penetrate into hard-to-reach areas and effectively eliminate pathogens [14,15]. This technology has gained traction in various settings, including healthcare facilities, due to its efficiency, safety, and versatility [13,16,17].

Compared with traditional disinfection methods, dry fog offers several advantages. Firstly, dry fog can be used to disinfect large areas quickly and effectively, reducing the time required for cleaning and decontamination procedures [17,18]. Secondly, the fine mist produced by dry fog can penetrate into small crevices and spaces, reaching areas that may be missed by conventional cleaning methods [17,18]. Finally, dry fog systems can be used to disinfect both surfaces and the air, providing comprehensive protection against the spread of pathogens [15,18].

Portable aerosol-producing machines, specifically those designed to generate disinfecting dry fog, are well known, have been available for a relatively long time in the market, and are widely used in different work settings. These machines spray dry fog from bottom to top (floor to ceiling) and can provide greater flexibility and mobility, allowing for easy movement and deployment in various hospital areas. This portability is particularly beneficial in settings where frequent relocation is necessary, such as between patient rooms or during temporary isolation measures. Additionally, portable machines can be used in confined spaces or areas with limited access, such as small examination rooms or isolation cubicles. However, portable machines may have limitations in terms of coverage area and the ability to maintain a consistent dry fog distribution.

Mounted machines, on the other hand, are fixed in place and spray dry fog from top to bottom (ceiling to floor). These machines often offer superior coverage and distribution capabilities, as they can be strategically positioned to optimize aerosol dispersion throughout hospital wards and other large spaces. Mounted machines are particularly suitable for high-traffic areas or areas with high ceilings, where they can effectively reach all corners of the environment. Additionally, mounted machines can be integrated into existing ventilation systems, enhancing the efficiency of dry fog delivery and reducing the risk of uneven distribution. However, mounted machines lack the portability of portable units and may be more challenging to install and maintain in certain hospital settings.

In this study, we explored the novel application of a mounted dry fog disinfection system in a large-scale open inpatient ward. While dry fog technology has been traditionally used in more controlled environments such as laboratories or operating theatres, data from the usage of such systems (to the best of our knowledge) as a routine disinfecting methodology and their effect on microbial burden in an uncontrolled environment (such as an inpatient ward of an internal medicine department) are not available. Despite the challenges associated with implementing a mounted system, proof of its efficacy in an uncontrolled environment, confirmation of the system’s safety and optimal maintenance, and results demonstrating its potential to reduce microbial burden are extremely valuable and can greatly contribute to a safer healthcare setting.

## 2. Results

A series of environmental surface and air samples were collected from all rooms included in the study before the installation of the system to determine the basal contamination level in the ward as the initial control measure. The effect of dry fogging on the reduction in microbial burden was evaluated in two ways. Firstly, we determined the “reduction effect” of dry fogging using the overall microbial count in treated rooms by measuring the significance of changes in trends of microbial isolations in these rooms (before vs. after). Secondly, we evaluated if such changes were still significant by comparing the results among different room types and between these room types and the controls; this was called the “degree of reduction”. This was because, although the reduction effect might be significant, the differences in disinfecting schedules among different room types might cause a decrease in the degree of reduction. Although the use of the system can cause a meaningful decrease in the total number of isolated colonies, that reduction might not be significant in specific room types (due to their different disinfecting schedules) in terms of the overall microbial burden in comparison with other rooms. The level of reduction was measured by comparing changes in trends of isolation from rooms where dry fogging was used among each other and the controls.

A total of 183 environmental surface swabbing samples and 14 samples for the determination of air microbial contamination (2 × 7 sampling processes with total sampling of 14,000 L of ward air) were collected during six scheduled tests. The presence of aerobic and anaerobic bacterial and fungal colonies was tested on solid and liquid culture media from the collected samples.

### 2.1. Effect of Dry Fogging on the Reduction in Aerial Microbial Contamination

**Figure 1:**
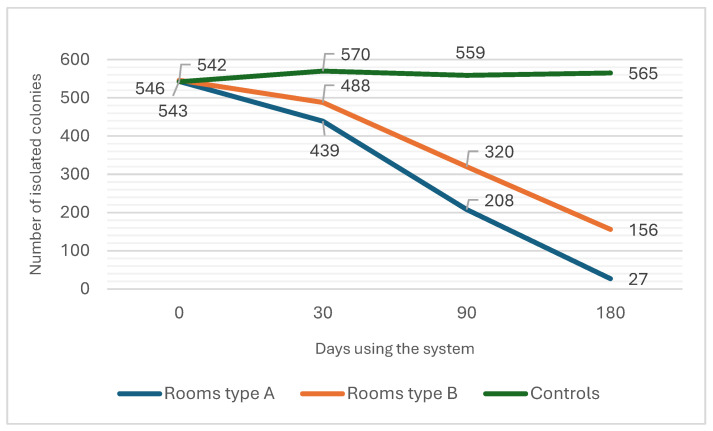
Effect of dry fogging on changes in the ward air microbial burden (in terms of colony isolation) and the difference in efficacy between disinfecting schedules (room types) and the control.

A total of 1631 (bacterial and fungal) colonies were isolated from air samples during the testing of the basal contamination level from all three-room types. The number of colonies isolated from air samples in relation to the usage of the system was consequently tested 30 days (first test), 90 days (second test), and 180 days (third test) after the basal contamination level test. In room type A, 439 colonies were isolated in the first test, 208 colonies were isolated during the second test, and 27 colonies were isolated during the third test (Figure 1). In room type B, 488 colonies were isolated during the first test, 320 colonies were isolated during the second test, and 200 colonies were isolated during the third test. In control rooms, the number of isolated colonies stayed steadily over 540 (namely, 570, 559, and 565) during each subsequent test (Figure 1).

To assess the system’s reduction effect, we conducted a combined analysis of room type A and room type B over time as a single group using a mixed-effect model analysis. The model revealed a significant decline in values over time, with a time coefficient of −155.85 (SE = 19.192, *p* < 0.001), reflecting a strong downward trend across both rooms as a result of the system’s impact. The intercept for the baseline was 730.5 (*p* < 0.001), indicating an initially high starting point. This analysis confirms that, collectively, room type A and room type B experienced a marked decrease in aerial microbial contamination, suggesting the system’s effectiveness in reducing the aerial microbial count over time.

To evaluate the degree of reduction in the system, we used a pairwise mixed-effect model, comparing the changes in aerial microbial contamination in each room type with each other and with that of the control. Room type A exhibited a significantly higher baseline than that of the control, with a coefficient of 204.5 (SE = 64.484, *p* = 0.002), and a distinct downward trend, indicated by the time-room A interaction term (coefficient = −183.7, SE = 18.239, *p* < 0.001). Room type B also had a significantly higher baseline than that of the control, with a coefficient of 167.5 (SE = 68.343, *p* = 0.014), and it showed a significant but more moderate decline over time (time-room B coefficient = −139.6, SE = 19.33, *p* < 0.001). When directly comparing room type A with room type B, both rooms exhibited significant declines (time coefficient = −177.9, SE = 18.038, *p* < 0.001), though the rate of decline was not significantly different between them (time-room B interaction coefficient = 44.1, SE = 25.51, *p* = 0.084). These results indicate that, while both rooms experienced substantial reductions in values, room type A’s trend was slightly steeper. This underscores that the system’s usage caused a significant degree of reduction in both disinfecting schedules and imposed a meaningful decrease in the aerial microbial burden.

### 2.2. Effect of Dry Fogging on the Reduction in Surface Microbial Contamination

Evaluation of changes in surface microbial contamination and proof of the system’s effect on such changes were rather challenging. This was firstly due to the highly contaminable surfaces of the wardrooms and the extent of uncontrollable variables that could cause such contamination. To solve this problem, we opted to evaluate the holistic long-term effects of the system on changes in surface contamination (if any) in treated rooms vs. the controls rather than using the point prevalence control of the results (before vs. after cleaning).

**Figure 2:**
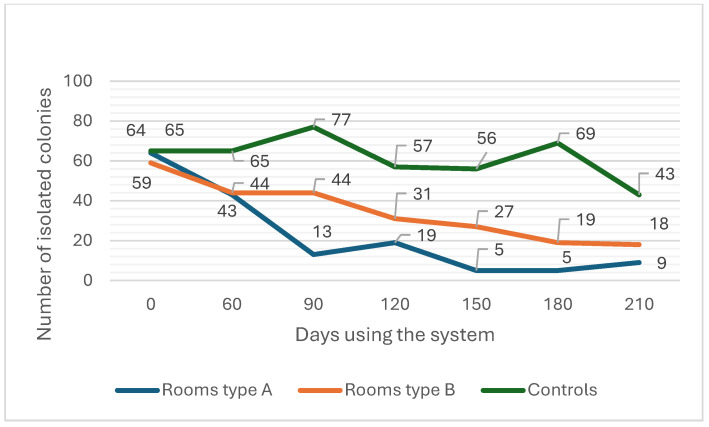
Effect of dry fogging on changes in ward rooms’ surface microbial burden (in terms of colony isolation) and the differences in efficacy between disinfecting schedules (room types) and the control.

Secondly, the microbiological results from surface samplings are presented in qualitative form (example: presence of massive bacterial growth or moderately massive fungal growth) rather than quantitative form, limiting the statistical evaluation of such results. To address this problem and for the possibility of statistical evaluation of environmental surface samples, on the basis of qualitative cultural growth, we quantified the results by means of numerical presentation. The presence of any microbial colonies was delineated by one point. In the case of the presence of massive cultural growth (of a bacterium or a fungus (saprophytes, hyphae) in a sample), three additional points were added. In the case of moderately massive cultural growth, two additional points were added, and in the case of minor cultural growth, one additional point was added to the given sample. With this method, every sample received a point as a numerical presentation of the presence or absence of microbial colonies, which included the extent of such presence at the same time. Consequently, we added all the numerical presentations of all the samples collected from a room during a scheduled sampling to calculate the quantitative microbial burden of that room (Figure 2).

Six subsequently scheduled surface samplings were performed in all three-room types (Figure 2). The places at which samples were collected were recorded for each room to evaluate their relevance to the contamination of patients/staff and for pattern monitoring. As with the air sampling, the reduction effect and the degree of reduction due to dry fogging were evaluated in terms of changes in surface microbial contamination in relation to the long-term use of the system (using the numerical presentation).

**Figure 3:**
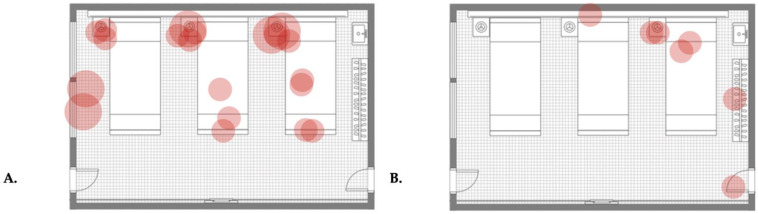
Map plot of the qualitative colony isolation from the first (**A**) and last (**B**) surface sampling of a room type A. Each figure presents a ward room where three patients’ beds (bed number 1: right, bed number 2: in the middle, and bed number 3: left), patients’ closets with hangers, sinks (both on the right), bed tables (on the left side of each bed), bed lamps and monitors (on top of each bed), two windows (on the left), the entrance door (right bottom), and the balcony door (left bottom) are distinguishable. Each red circle demonstrates an isolation. Small circles represent minor cultural growth, moderate circles represent moderately massive cultural growth, and large circles represent massive cultural growth for each isolation. Labelling of room furniture and a guide for identifying indicators of cultural growth are presented in detail in Figure S1.

**Figure 4:**
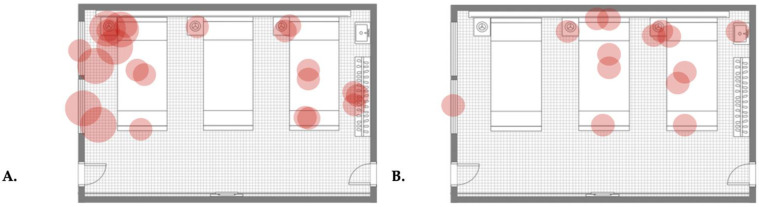
Map plot of the qualitative colony isolation from the first (**A**) and last (**B**) surface sampling of a room type B. Each figure presents a ward room where three patients’ beds (bed number 1: right, bed number 2: in the middle, and bed number 3: left), patients’ closets with hangers, sinks (both on the right), bed tables (on the left side of each bed), bed lamps and monitors (on top of each bed), two windows (on the left), the entrance door (right bottom), and the balcony door (left bottom) are distinguishable. Each red circle demonstrates an isolation. Small circles represent minor cultural growth, moderate circles represent moderately massive cultural growth, and large circles represent massive cultural growth for each isolation. Labelling of room furniture and a guide for identifying indicators of cultural growth are presented in detail in Figure S1.

**Figure 5:**
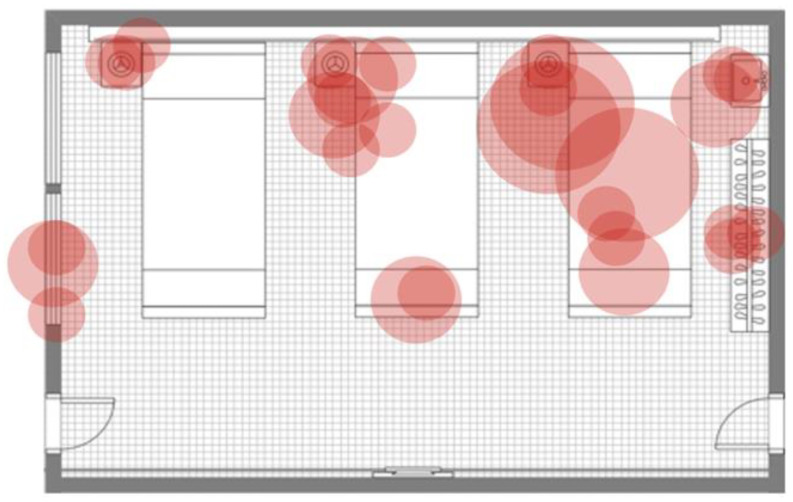
Map plot of the qualitative colony isolation from the first surface sampling of the control room. Each figure presents a ward room where three patients’ beds (bed number 1: right, bed number 2: in the middle, and bed number 3: left), patients’ closets with hangers, sinks (both on the right), bed tables (on the left side of each bed), bed lamps and monitors (on top of each bed), two windows (on the left), the entrance door (right bottom), and the balcony door (left bottom) are distinguishable. Each red circle demonstrates an isolation. Small circles represent minor cultural growth, moderate circles represent moderately massive cultural growth, and large circles represent massive cultural growth for each isolation. Labelling of room furniture and a guide for identifying indicators of cultural growth are presented in detail in Figure S1.

For a better understanding of the quantification (point assignment) based on qualitative growth, the evaluation of the first and last results from the surface sampling of room types A and B and the first result from a control room are presented in Table 1, Table 2, Table 3, Table 4 and Table 5. Similarly, for better demonstration of qualitative changes in the surface microbial burden (pattern monitoring and monitoring of differences in the presence and extent of cultural growth), map plots of isolated colonies from the same tests are presented in Figure 3, Figure 4 and Figure 5.

Pattern monitoring was conducted using an artificial intelligence application that controlled the sampling locations in the same rooms throughout the study period. The reason for monitoring was to determine the possibility of sampling bias (meaning several samplings from one place and no samples from other places). This monitoring allowed the authors to optimize the randomness of the data from the collected samples. In cases where more than three samples were collected from the exact same place in the same room in three different scheduled samplings, the application selectively removed one result with the least impact on the overall points from one of the time points.

In the combined analysis (reduction effect) of room types A and B over seven tested time points, the mixed-effect model revealed a significant overall decrease in the surface microbial burden. The time coefficient was −5.161 (SE = 2.002, *p* = 0.010), indicating a statistically significant downward trend across room types A and B in combination over the study period. The intercept was estimated at 45.643 (SE = 17.450, *p* = 0.009), reflecting the average initial burden in these rooms.

This result suggests that, collectively, room types A and B experienced a significant reduction in surface microbial burden over time, likely reflecting the impact of the system in these environments. The negative time coefficient confirms a consistent decrease in values across the sampling periods, supporting the effectiveness of the system in reducing the surface microbial burden when considering these two rooms together.

However, once we begin to compare the degree of reduction due to dry fogging by comparing the changes over time with that of the controls, despite the negative trends in both room types, the result is not statistically significant. The model includes time as a fixed effect to assess any overall downward trends in microbial burden, and the room type (A, B, and control) is used as a factor to identify differences in response to the system. The model’s intercept was 71.57 (SE = 16.905, *p* < 0.001), indicating a high initial burden level, with a similar starting point across all three room types (room type A: coefficient = −13.43, *p* = 0.574; room type B: coefficient = −38.43, *p* = 0.108). Rationally, the main effect of time was not statistically significant (coefficient = −2.821, SE = 2.440, *p* = 0.248) when the control rooms were included in the model. However, the time-room interactions provide valuable insights into the distinct trends for room types A and B. Specifically, the time-room type A interaction approached significance (coefficient = −6.071, SE = 3.269, *p* = 0.063), suggesting a potential downward trend in room type A that was stronger than that in the controls, though it did not reach full significance. In contrast, room type B did not show a statistically significant interaction with time (time-room B: coefficient = 1.393, SE = 3.451, *p* = 0.687), indicating that the changes in room type B over time were not distinct from the controls. This suggests that, while both room type A and room type B started with similar microbial burden levels, only room type A showed a possible trend toward a decreasing microbial burden over time. This model points to a differential effect of the system across room types, with room type A exhibiting a stronger response than room type B. Although these results suggest a potential benefit in room type A, further testing with a larger sample or additional time points could clarify these trends.

### 2.3. Effect of Dry Fogging on Changes in Types of Surface Microbes Isolated

**Figure 6:**
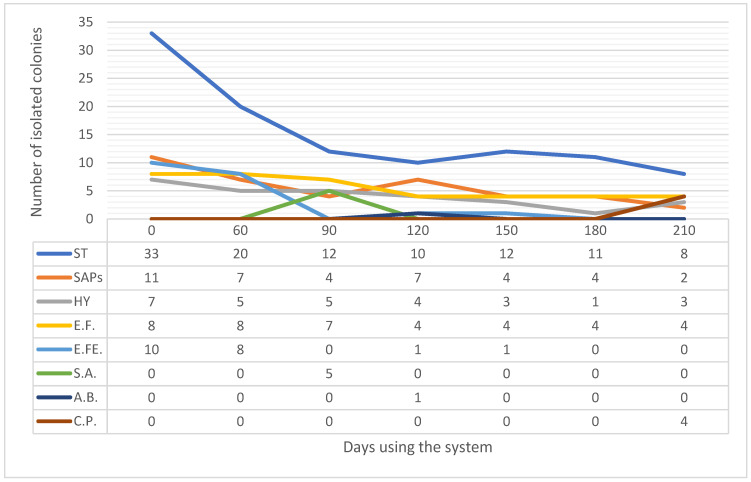
Trends of isolation of all microorganisms sampled from room types A and B using environmental sampling throughout the study. ST: Coagulase-negative *Staphylococcus* species, SAPs: aerial saprophytes, HY: hyphae, E.F.: *E. faecium*, E.FE.: *E. faecalis*, S.A.: *S. aureus*, A.B.: *A. baumannii*, C.P.: *C. perfringens*.

For this evaluation, we recorded data on all isolated samples, including the names of bacteria and types of microorganisms (fungi or bacteria), across the timespan of surface samplings from each room type. By organizing the data on isolation frequencies, we identified the most frequently occurring and sporadically appearing organisms in each room. Unsurprisingly, the most commonly isolated microorganisms were coagulase-negative *Staphylococcus* species (CoNS), followed by aerial saprophytes (SAPs), hyphae (HY), *Enterococcus faecium*, and *Enterococcus faecalis* (Figure 6). In contrast, *Acinetobacter baumannii*, *Staphylococcus aureus*, and *Clostridium perfringens* appeared only sporadically. By plotting the data collected on the number of isolations across seven environmental samplings (from rooms where the system was used), we evaluated the trends in isolation counts to determine whether there was a significant decrease in the presence of various microorganisms over time (Figure 6). To evaluate trends over time, we applied Kendall’s Tau correlation analysis, a non-parametric statistical test that is particularly suited for detecting monotonic trends without assuming a specific data distribution. Given the small sample size and potential nonlinear patterns in the isolation counts, Kendall’s Tau was chosen over linear regression, as it provides a robust approach for analysing data with limited points and varying patterns without forcing a linear fit.

Coagulase-negative *Staphylococcus* species, SAPs, HY, *E. faecium*, and *E. faecalis* all demonstrated statistically significant negative trends in isolation counts over time, with Kendall’s Tau values near −1, indicating a strong, consistent decrease in microbial presence on surfaces. The associated *p*-values for these organisms (CoNS: *p* = 0.015, SAP: *p* = 0.017, HY: *p* = 0.009, *E. faecium*: *p* = 0.017, and *E. faecalis*: *p* = 0.039) were below the 0.05 significance level, supporting the conclusion that the observed downward trends were statistically meaningful. Randomly isolated microorganisms were not tested due to their sporadic isolation pattern. This finding is in accordance with the data on the reduction effect on surface microorganism contamination due to dry fogging presented earlier, showing a significant decrease in the combined number of isolations in rooms where the system was used. Additionally, these data demonstrate that the effect of dry fogging included all types of frequently isolated microorganisms.

Through a very broad qualitative comparison using the data presented in the map plots of the first and last samplings in room types A and B and the control (Figure 3, Figure 4 and Figure 5), we can distinguish the changes in qualitative growth of isolated samples. For example, we can compare the data in Figure 3, which demonstrates the surface samplings in room type A on the 30th day after the initiation of the disinfecting schedule, with those in Figure 4 (which shows the qualitative growth in room type B) and Figure 5 (which demonstrates the qualitative growth in a control room on the same sampling date). No massive cultural growth of any microorganism was documented in either room type A or room type B in comparison with the control, demonstrating the effect of the system (after 30 days of usage) on changes in qualitative growth among the rooms. Similarly, a comparison of Table 1 with Table 2 (Figure 3) or Table 3 with Table 4 (Figure 4) (meaning the first sampling vs. last sampling in the same room type) shows a decrease in the number of isolations, an increase in the number of sterile samples, and a change in moderately massive cultural growth to minor cultural growth in both of the room types. These data present the effect of dry fogging on the gross qualitative changes observed when comparing cultural growth between different time points in different room types.

## 3. Discussion

The benefits of dry fogging as a disinfection method in healthcare settings are well documented, showcasing its effectiveness in reducing microbial contamination and infection risks. For instance Emerald et al. demonstrated that dry fogging (using Sanosil as a disinfectant) outperformed glutaraldehyde in disinfecting ventilator tubes, demonstrating its potential to lower ventilator-associated pneumonia risks [19]. In a publication by Coughlin et al. authors revealed a significant decrease in illness-related absenteeism following dry fogging implementation in a paediatric daycare centre, highlighting its broader public health impact [20]. Research by Patel et al. further confirmed dry fogging’s ability to reduce bioburden on critical care surfaces, demonstrating its effectiveness in enhancing environmental cleanliness [21].

In comparison, our study offers a distinct perspective by evaluating the long-term efficacy of dry fogging in open inpatient wards with high microbial reintroduction rates, a context largely unexplored in prior studies. Unlike earlier research conducted in more controlled or specific settings, we demonstrated that dry fogging significantly reduced airborne microbial loads in high-traffic healthcare environments over extended periods. Our data combine analyses of airborne and surface contamination, providing robust evidence of the system’s sustained impact despite dynamic contamination pressures.

### 3.1. Efficacy of Dry Fogging in Airborne Contamination Control

The primary goal of the study was to determine whether scheduled air disinfection through dry fogging in an open inpatient wardroom where the microbial contamination/reintroduction level is very high causes a meaningful decrease in aerial microbial burden. This is especially relevant when the containment of a microenvironment strategy (by having separate rooms with doors that are closed for 24 h) is not achievable. Our data demonstrated that scheduled dry fogging can significantly decrease the overall microbial contamination level in the air. Evaluation of both the reduction effect and the degree of reduction when using the system showed a significant decrease in the number of microbial isolations. This result leads to two important conclusions. Firstly, using dry fog is superior to using no air microbial disinfection (room type A vs. control: SE = 64.484, *p* = 0.002; room type B vs. control: SE = 68.343, *p* = 0.014). This observation aligns with results from published studies (such as that of Otter et al.) emphasizing the insufficiency of management of passive air decontamination in high-occupancy or high-traffic healthcare environments where pathogens are constantly reintroduced [22]. Secondly, our data indicate that scheduled air disinfection holds significance even in a high-microbial-load environment, such as an open internal medicine inpatient ward (SE = 19.192, *p* < 0.001). This is a novel finding. Our result indicates that air disinfection in an open, high-traffic room, even when contamination levels remain high (due to ongoing entry and activity), can still significantly decrease the overall microbial load (time coefficient of −155.85 (SE = 19.192, *p* < 0.001)). In this sense, disinfection in such environments, while not eliminating contaminants entirely, can still lower the baseline microbial load and reduce the concentration of airborne pathogens momentarily, thereby limiting the potential for transmission. Reducing exposure to harmful microorganisms is especially important in healthcare settings with a high burden of nosocomial airborne contaminations. Studies by Sattar et al. and Weber et al. demonstrate that even intermittent air disinfection can decrease microbial burden temporarily, which can be especially beneficial in reducing exposure to pathogens for vulnerable individuals and can work as a valuable supplementary control measure, helping to mitigate some contamination despite ongoing reintroduction [22,23,24]. Although it is challenging to maintain complete air decontamination in open spaces, regular disinfection cycles can still play a critical role in maintaining lower microbial levels overall, providing an additional layer of protection against airborne infections.

Another important finding is the effect of the exposure time and the disinfection cycle’s duration on the efficacy of microbial reduction. This is especially apparent when comparing the last two aerial control samples. With the same concentration of the disinfecting solution and the same disinfecting schedules, the results from both room types showed a notable decrease in the isolation of aerial microbes upon only increasing the exposure time and disinfecting cycle (DC) duration (Figure 1) between phase 2 and phase 3. The observed variability in microbial reduction between room types A and B highlights the dynamic challenges associated with applying dry fogging systems in open healthcare environments. Beyond the differences in disinfection frequency, other unmeasured factors, such as variability in room occupancy, differences in patient and staff activity levels, and potential disparities in air circulation, may have influenced the outcomes. These findings suggest that while frequency of application is critical, the effectiveness of dry fogging systems may also depend on other contextual factors unique to each room. This raises important questions about the role of environmental heterogeneity and operational workflows in determining the success of such systems. Future research could explore how detailed environmental monitoring and workflow analyses might further elucidate the impact of these variables, leading to optimized protocols for consistent microbial reduction across different room types.

### 3.2. Surface Contamination: Evaluating the System’s Limitations

This study’s findings regarding surface microbial contamination reveal an interesting dichotomy between observed declines within treated rooms and comparisons with control rooms. Within each treated room, microbial isolation counts on surfaces significantly decreased over time, reflecting the system’s effectiveness in reducing surface microbial burden. However, when these results are compared with those of the control rooms, the decline is no longer statistically significant. This discrepancy can likely be attributed to several uncontrollable factors that are unique to surface contamination in active healthcare settings, such as constant patient and staff contact, which regularly reintroduces microorganisms onto surfaces.

This limitation of the dry fogging system for surface contamination is consistent with findings in the literature. In a published study, Boyce et al. emphasize that surface contamination in healthcare environments is particularly challenging to manage due to high levels of interaction with various surfaces and frequent microbial reintroduction by people and equipment [25,26]. This research suggests that routine cleaning protocols must be enhanced with adjuvant disinfection systems to maintain low microbial levels on high-touch surfaces [26]. Another factor contributing to the non-significant results may be the speed of recontamination of healthcare facilities’ surfaces [27]. Microbial recontamination occurs rapidly after cleaning, particularly in high-occupancy healthcare areas [13,28]. In a study by Salgado et al. the authors found that standard disinfection protocols reduced microbial counts temporarily, but significant microbial presence was often detected again within hours of cleaning due to environmental factors, patient interaction, and staff activities [29].

In our study, while the dry fogging contributed to lower microbial counts within the treated rooms, the lack of a lasting effect when compared with the control highlights the need for continuous intervention on surfaces exposed to constant contamination. This highlights that, as a disinfecting process, dry fogging should be used only as an adjuvant method for surface cleaning. This is similar to the findings of a recent publication highlighting that, while systems such as dry fog can contribute to overall cleanliness, they cannot fully replace hands-on cleaning, especially on surfaces with frequent human contact, which acts as a constant source of contamination [25].

### 3.3. Importance of the Dry Fogging System as an Adjuvant for Routine Cleaning

Despite these limitations, our findings demonstrate that dry fogging provides measurable benefits when used as an adjunct to routine cleaning practices. While it cannot achieve sterile surfaces alone in an open ward setting, it adds value by reducing the overall microbial burden on surfaces within the treated rooms over time. The system’s daily use in rooms of type A proved particularly effective, suggesting that increased frequency enhances its impact on microbial control. It is noteworthy to mention that the results from surface sampling in rooms of type A, despite their lack of significance in comparison with the controls regarding the degree of reduction, still provided a very notable decrease in the isolation count (coefficient = −6.071, SE = 3.269, *p* = 0.063), nearly approaching the level of significance. Moreover, the use of dry fogging demonstrated a long-term decrease in qualitative cultural growth of different microbial colonies and frequently isolated microbes regardless of the pattern of sampling. By providing additional control, the dry fogging system aids routine disinfection methods and helps maintain cleaner environments in high-risk areas.

Our results further align with research emphasizing the need for multimodal disinfection strategies in healthcare environments [30,31]. Huang et al. advocate for integrating adjuvant disinfection systems, such as dry fogging, with regular cleaning protocols to maximize microbial control in healthcare settings [32]. This approach is particularly effective in environments where traditional methods struggle to address the rapid reintroduction of contaminants. Huang et al.’s research suggests that systems such as Hydrofogg™ can be highly effective in reducing baseline microbial loads on surfaces and in the air, serving as a proactive measure to complement hands-on disinfection [32].

The mounted system offered crucial advantages in our setting, especially given the need for large-scale disinfection within a limited timeframe due to the ward’s busy schedule. The Hydrofogg™ system’s top-down spraying design significantly reduces the spraying time compared to portable systems that spray from the ground up. In those systems, the disinfectant must first reach a sufficient level of saturation in the air before it settles, which adds to the overall spraying duration. Additionally, the Hydrofogg’s central control panel allowed for automated and semi-automated disinfection of individual rooms or multiple rooms simultaneously, which was highly beneficial for disinfecting multiple rooms at once or scheduling room-specific disinfections in a ward setting.

The implementation of a dry fogging system for air and surface disinfection in hospital environments offers notable benefits but also presents significant operational challenges and financial considerations that warrant careful evaluation. These systems require trained personnel to ensure proper handling, as well as regular maintenance and quality control to maintain optimal performance and effectiveness. The need for scheduled testing to assess the system’s efficacy and to determine necessary optimizations adds to the operational costs of the system. Additionally, recurring costs, such as the use of certified disinfectant solutions such as Sanosil, contribute to the financial burden. For example, maintaining a daily disinfection schedule for two ~50 m^2^ rooms requires approximately 20 L of disinfectant every two months, equating to 5 L per room per month. This necessitates careful budget planning to account for such recurring expenses, especially considering the desired reduction in microbial burden and the cost-effectiveness of the system.

From a financial perspective, while the initial investment in the mounted dry fogging system and its consumables is higher compared to traditional methods, the system offers potential long-term economic advantages. These include consistent application, broad coverage, and reduced reliance on manual labor, which could justify the higher upfront costs over time. Since a detailed cost-benefit analysis was not within the scope of this study, future investigations should evaluate both the financial and operational implications of deploying such systems in high-demand hospital settings to better understand their overall economic viability and inform decision-making for infection control strategies.

In addition to financial considerations, there are several technical challenges associated with implementing a dry fogging system in dynamic hospital environments. For instance, the need to temporarily vacate rooms during the disinfection process presents logistical challenges in departments with high occupancy rates, limiting the availability of rooms for patient care. Scheduling disinfection is further complicated by the unpredictable nature of patient admissions, making it difficult to align disinfection protocols with the department’s variable and often urgent demands. These operational limitations underscore the complexities of deploying dry fogging systems in real-world healthcare settings.

Lastly, the influence of environmental variables, such as air circulation, temperature, humidity, and foot traffic, on microbial reduction was not independently assessed in this study. Given the nature of the open department setting, these factors were allowed to fluctuate naturally, reflecting the real-world conditions under which the system would be used. While this approach enhances the study’s practical relevance, it also introduces potential confounding variables that may impact the uniformity and effectiveness of microbial reduction. Future research should aim to evaluate these environmental factors in controlled settings to better understand their effects on disinfection efficacy. Including such assessments would provide valuable insights for optimizing the use of dry fogging systems in hospital environments.

Combining the results from the air and surface samplings indicates that room type A, with a disinfecting schedule of daily fogging with a 6% concentration of disinfectant media used over two cycles of 6 min of spraying, is superior to room type B and to the non-use of dry fogging, as it causes a significant decrease in airborne microbial burden, a significant decrease in the surface microbial count, and an improvement in the qualitative growth trends of microorganisms in an inpatient ward of an internal medicine department.

## 4. Materials and Methods

### 4.1. Healthcare Facility, Study Design, and Disinfection Protocols

In this prospective, interventional, double-blinded clinical trial, we evaluated the efficacy of dry fog implementation in reducing bacterial and fungal burden in an inpatient ward at the fifth Department of Internal Medicine, University Hospital Bratislava, Ružinov, from September 2023 to the end of March 2024.

The primary outcome was defined as a significant decrease in airborne microbial burden in the inpatient ward when using dry fog compared with a control.

Secondary outcomes were defined as proof of decreased surface and environmental microbial burden in the inpatient ward using dry fog in comparison with the control.

The Hydrofogg™ system was installed in six ward rooms, with a separated central panel, allowing automated or semi-automated control of defined disinfection cycles for each room. Hydrofogg™ is a hydraulically integrated system designed by Detectair Technologies, s.r.o. (Bratislava, Slovakia) [33], for humidifying and disinfecting indoor spaces using micro-droplets (dry fog) produced from water or a disinfectant solution as the medium. Sanosil S006 Ag (3%) and Sanosil S010 Ag (6%) (Sanosil AG, Hombrechtikon, Switzerland) were used as disinfectant media during the study period (composition: 5 g/100 g (5%) hydrogen peroxide CAS 7722-84-1, 0.01 g/100 g (0.010%) silver CAS 7440-22-4) [34].

The study was divided into four phases.

Initial phase (September 2023).

The initial phase was used to determine the minimum concentration of the disinfecting solution and the minimum duration of the DC needed for a meaningful decrease in the overall microbial burden. A series of tests were performed before (initial control measurements) and at the end of the initial phase to evaluate the efficacy of the predetermined DC duration and concentration of the disinfecting solution.

The reason behind the gradual changes in DCs and the concentration of the disinfecting solution was simply the novel characteristics of the study. The use of a mounted dry fog system in settings similar to those in our study has not been documented in published studies, and no data on the optimization of exposure conditions for dry fogging in an open inpatient ward are available. Hence, optimization based on the results and gradual correction of manipulable variables during the study time frame was the best approach possible. The initial DC was programmed as follows: 3 min (composed of repetitive cycles of 15 s of spraying followed by 10 s of standby, designated as the primary exposure time) using 345 mL of disinfectant, producing 34.5 cm^3^ of dry fog. The concentration of disinfectant media was initiated at 3% (lowest commercially available concentration).

The system was installed in six inpatient ward rooms, positioned sequentially along the same corridor, though each room had its own ventilation system. All rooms were uniform in size (48.5 m^2^) and had the same layout for patient beds and healthcare equipment, which included identical furniture and fittings. Each room contained three patient beds, a shared wardrobe, and a shared sink. Mounted above each bed were bed lamps and monitors for vital signs, while a bed table was positioned to the right of each bed. Each room featured two doors opposite each other: one leading to the corridor and the other to a balcony. Additionally, each room had two large, openable windows facing the balcony (see Figure 3, Figure 4 and Figure 5 for a room layout map). The Hydrofogg™ system was installed on the wall opposite the beds, designed to spray disinfectant towards them. In each room, the system consisted of a single looped tube, with three nozzles positioned on the lower section of the tube to release the disinfecting mist. These tubes were mounted 10 to 30 cm below the ceiling.

Three room types (A, B, and control) were delineated during the study. For each room type, two rooms were designated to omit the environmental bias of a single room pattern. The only difference between each room type was the disinfecting schedule applied. The room type A comprised rooms in which the system was used once per day with a defined concentration of disinfecting solution and DCs in the given phase. Room type B comprised rooms in which the system was used once every third day (twice a week) with a defined concentration of disinfecting solution and DCs in the given phase. Control rooms comprised rooms in which the system was not used. The normal daily ward workflow was followed (patient discharge, patients’ admission, family visits) regardless of the disinfection schedules and room types. Day-to-day surface or environmental cleanings and sanitary disinfections of rooms (including surface disinfection twice a day using Incidin Oxyfoam S (Ecolab, Deutschland GmbH, Monheim am Rhein, Germany), Meliseptol Foam pure (B. Braun Melsungen AG, Melsungen, Germany), and Chloramine 0.2%/L solution) were performed based on the normal defined local schedules of the hospital regardless of the use of the system.

Environmental sampling was performed according to predetermined monthly schedules. During each sampling process, one room of each type was sampled. Except for the system operator (following the planned DC schedule), staff performing environmental sampling and diagnostic laboratory staff were blinded to the schedule of disinfections and room types to omit sampling/evaluation bias. The rooms from which samples were collected were intentionally changed during each sampling process to omit pattern recognition. During each DC, patients were moved out of the rooms, and the rooms were closed during the primary exposure time and left closed for another 45 min (based on instruction from the manufacturer) [33] after finishing the spraying cycles (designated as the actual exposure time and sedimentation time). After the end of the actual exposure time, the rooms were reopened, and the patients were moved back into their rooms. All of the patients’ belongings (except for food or drinks), medical instruments, and furniture were left in the rooms during each DC.

Phase 1: Protocol optimization (beginning of October 2023 until the end of November 2023).

The objective of phase 1 was to optimize the predefined settings of the initial phase and apply them for a period of two months to evaluate the effects of the system using these optimized settings. Based on the results of the tests in the initial phase, an insignificant decrease in the number of microbial isolations (compared with the initial control measures) was demonstrated, causing the increase in the DCs to 6 min (2 × 3 min of primary exposure time) without a change in concentration of the disinfecting solution at the beginning of phase 1.

**Figure 7:**
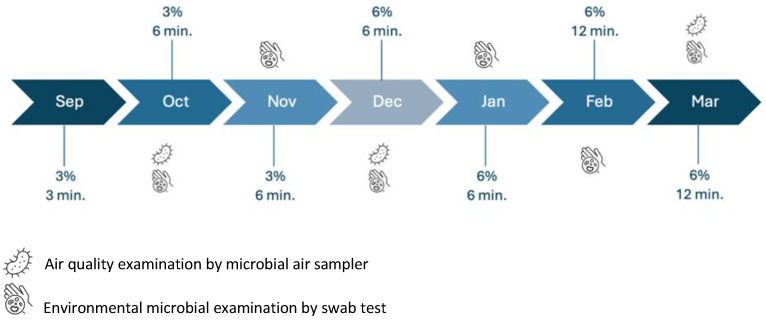
Diagram of changes in disinfection cycles (shown in minutes), the concentration of the disinfecting solution (shown in %), and a timetable of tests conducted throughout the study.

Although the gradual increase in DCs and primary exposure times was based on the results of the system’s efficacy, the maximum possible DC was determined at the beginning of the study based on the increase in the humidity level in the rooms in relation to the use of the system. This change in the humidity level could not exceed the threshold of 60%. Based on the size of the rooms in our department, 12 min was considered the maximum exposure time that could be planned (if needed) to reach the maximum efficacy (Figure 7).

Phase 2: Protocol refinement (beginning of December 2023 until the end of January 2024).

Based on results of the tests in phase 1 and the control tests at the beginning of phase 2, the authors decided to increase the concentration of the disinfecting solution. The changes in the concentration of disinfecting solutions could be applied if a new aerial sample from one room type resulted in a reduction in microbial count of less than 50% in comparison with the previous test, given that the exposure time was kept the same. After two months of DCs of 6 min with a 3% concentration (continued from the initial phase), the new results demonstrated the need to increase the concentration of the disinfecting solution to 6%. By the beginning of phase 2, a declining dynamic in the concentration of microbial isolates was observed, especially in rooms of type A, implying the efficacy of the system. However, the stagnation of results in rooms of type B and the insignificant reduction in the microbial isolation level when comparing the last samplings presented the need for refinement of the protocol.

Phase 3: Final refinement (beginning of February 2024 until the end of March 2024).

Considering the already apparent efficacy of the 6% concentration of the disinfecting solution from the last sampling tests, the authors decided to increase the DC to the maximum determined level of 12 min by increasing the exposure time from 3 min to 6 min (composed of repetitive cycles of 20 s of spraying followed by 10 s of standby) using 690 mL of disinfectant, producing 69 cm^3^ of dry fog, and the DCs were increased to 2 × 6 min cycles to determine the dependency of the microbial burden on exposure during the last two months of the study. The final sampling tests were performed in March 2024, marking the end of the study.

### 4.2. Sampling Protocol and Proof of Bacterial Strains

Environmental sampling was performed using sterile swabs from surfaces in ward rooms. The sampling locations and number of swabs were decided by sampling staff from the department of hospital hygiene and epidemiology, normally including a minimum of 10 environmental samples collected from different places in each examining room. Samples were processed using conventional methods approved by the Slovak Ministry of Health. Bacterial identification was performed with standard bacterial culture growth on solid media using Columbia blood agar (CBA) with 7% sheep blood (EnviroLab s.r.o., Bratislava, Slovakia) and thioglycolate broth as liquid media for evaluation of the presence of aerobic and anaerobic microorganisms. Strain identification was performed using conventional methods and matrix-associated laser desorption/ionization time-of-flight mass spectrometry (MALDI-TOF MS) (Merck, Darmstadt, Germany) where applicable [35].

An SAS Super IAQ microbial air sampler (P. No. 17-D-12321, Avantor^®^, VWR, Radnor, PA, USA) was used for control of airborne microbial burden and ward air quality, with a flow rate of 100 L/min. Sampling was conducted in 10 min intervals (1000 L of sampled air from each room), and inoculation was performed in two different culture media. CBA and Sabouraud agar (SAB) (with 2% glucose and chloramphenicol (EnviroLab s.r.o.)) were used for the microbial inoculation of sampled air. The sampling protocol, exposition time, sample placement, and culture handling were performed and controlled by staff from an environmental microbial evaluation laboratory (Medirex a.s., Nitra, Slovakia) and the department of hospital hygiene and epidemiology, following standardized operational protocols. The optimal environmental conditions required by the sampler’s manufacturer were met during each sampling session [36]. The CBA-inoculated cultures were incubated at 35 °C for 24–48 h, and the SAB-inoculated cultures were incubated at 25 °C for 5–7 d based on conventional methods approved by the Slovak Ministry of Health. Cultivation analysis, microorganism concentration evaluation, and strain identification were performed based on WHO assessment of exposure to indoor air pollutants [37] and in accordance with the European harmonization framework for urban air, indoor environments, and human exposure (EUR 14988 EN) [38].

### 4.3. Statistical Analysis

Data collected during the monitoring period were analysed using Microsoft Excel (version 16.88). The association between the decrease in the airborne/environmental microbial burden and the use of the system was analysed using a mixed-effect model and a paired mixed-effect model. The time effect refers to the overall trend in microbial isolation counts across the sampling points, regardless of the room. This effect is aimed at capturing whether microbial counts decrease over time due to the system’s use, independent from specific room conditions or usage frequency. On the other hand, the time-room effect (also known as the interaction effect between the time and room) examines whether the trend over time differs between the two types of rooms (room type A and room type B) based on the system’s different usage patterns. This interaction term allows us to observe whether the decline in microbial counts is stronger or weaker in one room than in another, indicating room-specific responses to the system’s application frequency. In this context, the time effect would be considered a fixed effect because we are specifically interested in the effect of the sequential days and how microbial counts change across these particular controlled time points. Similarly, the time-room interaction is also treated as a fixed effect, as it represents an explicit hypothesis about the differential effect of the system’s usage pattern between the two rooms; it is not randomly assigned but rather systematically applied.

For the analysis of the significance of changes in trends of isolations, we used Kendall’s Tau correlation analysis with *p* = 0.05 as the level of significance.

### 4.4. Ethical Consent

Ethical review and approval were not required for this study (as an interventional environmental study) because no direct examinations or interventions were applied to humans, in accordance with local legislation and institutional requirements.

## 5. Conclusions

This study demonstrates that scheduled dry fogging in an open inpatient ward with a high microbial load effectively reduces both airborne and surface microbial contamination, even in environments where complete containment is impractical. Our findings indicate that regular disinfection, particularly with extended exposure times, significantly lowers microbial presence, offering a meaningful reduction in potential pathogen transmission. While dry fogging cannot replace traditional surface cleaning due to rapid recontamination, it provides valuable support as an adjunct method, enhancing overall hygiene and contributing to infection control in high-traffic healthcare settings.

Further research is needed to fully evaluate the long-term efficacy and cost-effectiveness of mounted dry fog systems in inpatient wards. Additionally, investigating the optimal placement and configuration of these systems within different ward layouts is essential for maximizing their impact. By addressing these areas, future studies can provide valuable insights into the role of mounted dry fog technology in improving infection prevention and control in healthcare facilities.

## Figures and Tables

**Table 1:** First surface sample results from room type A and the point assignment based on the presence of microbial colonies and the qualitative extent of cultural growth.

	Place of Sampling	Qualitative Aerobic Culture Growth	Bacterium	Fungal Growth	Fungus	Liquid Culture Growth	
Room type A	Bed 1	Minor growth	CoNS	Sterile	-	*E. faecium*	
	Bedtable 1	Moderately massive growth	CoNS	Sterile	-	*E. faecalis*	
	Bedrail 1	Minor growth	CoNS	Sterile	-	*E. faecium*	
	Bed 2	Minor growth	CoNS	Sterile	-	*E. faecalis*	
	Bedtable 2	Minor growth	CoNS	Sterile	-	*E. faecalis*	
	Bedrail 2	Minor growth	CoNS	Sterile	-	*E. faecalis*	
	Lamp 1	Moderately massive growth	CoNS	Minor growth	Hyphae	*E. faecalis*	
	Lamp 2	Moderately massive growth	Saprophytes	Minor growth	Hyphae	*E. faecalis*	
	Lamp 3	Minor growth	CoNS	Minor growth	Hyphae	*E. faecium*	
	Windowsill 1	Moderately massive growth	CoNS	Moderately massive growth	Saprophytes	*E. faecalis*	
Points		14	10	5	4	10	Total: 43

CoNS: Coagulase-negative *Staphylococcus* species.

**Table 2:** Last surface sampling results from room type A and the point assignment based on the presence of microbial colonies and the qualitative extent of cultural growth.

	Place of Sampling	Qualitative Aerobic Culture Growth	Bacterium	Fungal Growth	Fungus	Liquid Culture Growth	
Room type A	Bed 1	Sterile	-	Minor growth	Hyphae	CoNS	
	Bedtable 1	Sterile	-	Sterile	-	CoNS + A. saprophytes	
	Bedrail 1	Sterile	-	Sterile	-	No growth	
	Bed 2	Sterile	-	Sterile	-	No growth	
	Monitor bed 2	Sterile	-	Sterile	-	CoNS	
	Bedtable 2	Sterile	-	Sterile	-	No growth	
	Bedrail 2	Sterile	-	Sterile	-	No growth	
	Lamp 1	Sterile	-	Sterile	-	CoNS	
	Lamp 3	Sterile	-	Sterile	-	No growth	
	Hanger	Sterile	-	Sterile	-	Aerial saprophytes	
	Sink	Sterile	-	Sterile	-	No growth	
	Doorknob	Sterile	-	Sterile	-	CoNS	
Points		0		1	1	7	Total: 9

CoNS: Coagulase-negative *Staphylococcus* species.

**Table 3:** First surface sampling results from room type B and the point assignment based on the presence of microbial colonies and the qualitative extent of cultural growth.

	Place of Sampling	Qualitative Aerobic Culture Growth	Bacterium	Fungal Growth	Fungus	Liquid Culture Growth	
Room type B	Bed 1 Bedtable 1 Bedrail 1 Lamp 2 Bed 3 Bedtable 3 Bedrail 3 Monitor bed 3 - Hanger Windowsill 1 Windowsill 2 -	Minor growth Minor growth Minor growth - Minor growth Moderately massive growth Minor growth Moderately massive growth - Minor growth Moderately massive growth Minor growth -	CoNS CoNS CoNS - CoNS CoNS CoNS CoNS - CoNS CoNS CoNS + *E. faecium*	Sterile Sterile Sterile Minor growth Sterile Sterile Sterile Moderately massive growth Minor growth Moderately massive growth Moderately massive growth	- - - Hyphae - - - Saprophytes Hyphae Saprophytes Saprophytes	*E. faecium* *E. faecalis* *E. faecium* No growth *E. faecalis* *E. faecalis* No growth *E. faecium* - *E. faecium* No growth No growth -	
Points		13	11	8	5	7	Total: 44

CoNS: Coagulase-negative *Staphylococcus* species.

**Table 4:** Last surface sampling results from room type B and the point assignment based on the presence of microbial colonies and the qualitative extent of cultural growth.

	Place of Sampling	Qualitative Aerobic Culture Growth	Bacterium	Fungal Growth	Fungus	Liquid Culture Growth	
Room type B	Bed 1	Minor growth	CoNS	Sterile	-	CoNS	
	Bedtable 1	Sterile	-	Minor growth	Hyphae	CoNS	
	Bedrail 1	Sterile	-	Sterile	-	CoNS	
	Bed 2	Minor growth	CoNS	Sterile	-	CoNS	
	Bedtable 2	Minor growth	CoNS	Sterile	-	No growth	
	Monitor bed 2	Sterile	-	Minor growth	Hyphae	*E. faecium*	
	Bedrail 2	Sterile	-	Sterile	-	*E. faecium*	
	Lamp 1	Sterile	-	Sterile	-	Aerial saprophytes	
	Windowsill 1	Sterile	-	Sterile	-	*C. perfringens*	
	Sink	Sterile	-	Sterile	-	CoNS	
Points		3	3	1	2	9	Total: 18

CoNS: Coagulase-negative *Staphylococcus* species.

**Table 5:** First surface sampling results from a control room and the point assignment based on the presence of microbial colonies and the qualitative extent of cultural growth.

	Place of Sampling	Qualitative Aerobic Culture Growth	Bacterium	Fungal Growth	Fungus	Liquid Culture Growth	
Control	Bed 1	Massive growth	CoNS	Minor growth	Hyphae	E. faecium	
	Bedtable 1	Massive growth	CoNS	Minor growth	Saprophytes	*E. faecium*	
	Bedrail 1	Moderately massive growth	CoNS	Sterile	-	*E. faecium*	
	Bed 2	Minor growth	CoNS	Sterile	-	CoNS	
	Bedtable 2	Minor growth	CoNS	Minor growth	Hyphae	CoNS	
	Monitor bed 2	Minor growth	CoNS	Minor growth	Hyphae	CoNS	
	Bedrail 2	Moderately massive growth	CoNS	Sterile	-	*E. faecium*	
	Lamp 1	Massive growth	*E. faecium*	Sterile	-	*E. faecium*	
	Lamp 2	Moderately massive growth	CoNS	Moderately massive growth	Hyphae	*E. faecium +*	
	-	-	-	-	-	*A. baumannii*	
	Windowsill 1	Moderately massive growth	CoNS	Minor growth	Hyphae	*E. faecium*	
	Hanger	Minor growth	CoNS	Minor growth	Hyphae	CoNS	
	Sink	Moderately massive growth	CoNS	Minor growth	Hyphae	CoNS	
Points		23	12	9	8	13	Total: 65

CoNS: Coagulase-negative *Staphylococcus* species.

## Data Availability

Data from environmental samplings and the quality of air may be provided upon written request.

## Supplementary Materials

The following supporting information can be downloaded at: https://www.mdpi.com/article/10.3390/antibiotics13121187/s1: Figure S1: Labelling of rooms furniture and indicators of cultural growth.
