# Supplementary material for: Efficacy of Antimicrobial Dry Fog in Improving the Environmental Microbial Burden in an Inpatient Ward
Source: Antibiotics (Basel). 2024 Dec 6;13(12):1187. doi: 10.3390/antibiotics13121187 (PMC11672662; doi:10.3390/antibiotics13121187)
Supplement: Supplementary file 1 [file antibiotics-13-01187-s001.zip › antibiotics-3333305-supplementary.pdf]

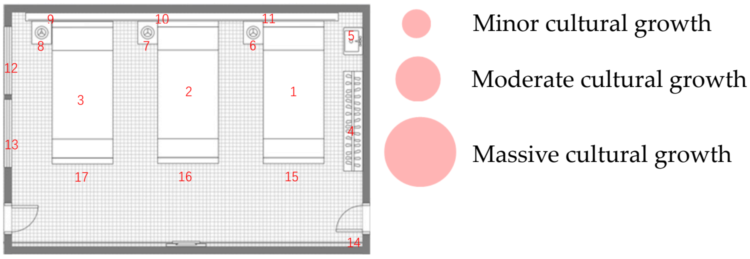

Figure S1: labelling of rooms furniture and indicators of cultural growth. 1: bed 1, 2: bed 2, 3: bed 3, 4: hanger, 5: sink, 6: bedtable and lamp 1, 7: bedtable and lamp 2, 8: bedtable and lamp 3, 9: monitor bed 3, 10: monitor bed 2, 11: monitor bed 3, 12: windowsill 2, 13: windowsill 1, 14: doorknob.
